# Understanding the plural landscape of cybersecurity governance in Spain: a matter of capital exchange

**DOI:** 10.1365/s43439-022-00069-4

**Published:** 2022-11-09

**Authors:** Cristina Del-Real, Antonio M. Díaz-Fernández

**Affiliations:** 1 Institute of Security and Global Affairs, Leiden University, The Hague, The Netherlands; 2 Department of International Public, Criminal and Procedural Law, University of Cádiz, Jerez de la Frontera, Spain

**Keywords:** Network governance, Nodal governance, Social network analysis, Delphi study, Interviews

## Abstract

This paper empirically explores the contribution and collaborative networks of public and private actors to cybersecurity provision in Spain. The article draws on data from three sources: policy and legal documents, a Delphi study with cybersecurity experts, and 34 interviews. Rooted in the theoretical underpinnings of nodal governance and anchored pluralism, the paper argues that the position of actors and public-private collaboration dynamics involved in cybersecurity governance can be understood through the analysis of capital exchange. Therefore, the study provides a list of the most relevant nodes for cybersecurity in Spain, assesses the capital they possess and how they exchange it through collaborative networks and explores the characteristics and barriers of these collaborative relationships. Analyses reveal that public organisations hold a preeminent position in cybersecurity governance despite large technology corporations’ greater economic and cultural capital. Remarkably, the paper identifies the central position of new public bodies in the network of cybersecurity nodes. Moreover, cultural barriers that are hindering public-private collaboration in Spain are identified. These results indicate that, despite the state’s difficulties in providing public solutions to cybersecurity challenges, Spain is an example of how governance can be anchored in public bodies through symbolic and social capital.

## Introduction

For a short period of time in history, the provision of security in Western societies was monopolised by two main actors: the Army and the Police. However, the complexity of current security problems has rendered the system established over the past two centuries outdated. New atomised threats, such as cyber threats, have overturned security provision. The conclusion that the state could no longer be the only provider of security was further fanned by the advent of neoliberalism in the 1980s. Among other effects, the security governance landscape has become crowded with new actors. The literature refers to this process as the ‘pluralisation of security provision’ [26]. Now, security is provided through interactive processes of resource exchange and collective negotiation of objectives among public and private actors, denominated as ‘governance’. And while the security governance literature is growing [3, 18], so far, it is more dominated by normative assumptions about how governments and private companies design security governance arrangements than by empirical examinations [33].

Cybersecurity governance’s lack of empirical scrutiny is even more accentuated. The anonymity and accessibility of cyberspace are a challenge for state security structures. The present study extensively analyses the public and private stakeholders involved in the Spanish cybersecurity landscape. The research design adopted the ‘pluralisation of policing’ theoretical lenses. Behind this generic concept, one can find two main approaches. On the one hand, some scholars adopt empirical approaches to understand security provision under the nodal governance paradigm [26]. This paradigm describes the non-monopolistic provision of security by multiple interrelated actors—called ‘nodes’—which can be of public, private or hybrid in nature, and which create governance networks through formal and informal resource exchange. Subsequent theoretical and empirical developments conceptualised this resource exchange through five forms of capital [19], based on Bourdieu’s forms of capital as units of exchange [5].

On the other hand, the anchored pluralism perspective justifies the normative assumption that the state is the only actor legitimised to provide security [29]. This perspective defends that the state must ‘anchor’ the plurality of security actors to safeguard the public interest [42, p. 93]. Subsequent work tested the validity of this approach in explaining the structures of security governance networks [33]. This paper explores the validity of nodal governance, capital exchange, and anchored pluralism assumptions to understand the structure and collaborative relationships between cybersecurity nodes in Spain.

There were three reasons for our decision to select Spain as a case study. First, because of Spain’s commitment to cybersecurity, highlighted by the Global Cybersecurity Index (GCI)—designed and published by the International Telecommunications Union [23]. According to this report, Spain scored fourth best in the world and third best in Europe. Second, it provides an opportunity to test the validity of the nodal governance paradigm outside the Anglosphere in which this paradigm originated. And finally, Spain is the European country with the highest level of administrative decentralisation [17]. Therefore, the landscape of nodes and the resources they own and exchange through collaboration relationships may vary from other countries due to Spain’s territorial organisation.

This paper is structured as follows. Sect. 2 presents a description of the Spanish cybersecurity system according to the law. In Sect. 3, the paper describes the research design, which consists of a Delphi study in three rounds and 34 interviews with cybersecurity experts. The study results are presented in Sect. 4 and then discussed in Sect. 5. This section also includes the study’s limitations and the proposal for future lines of research.

## An overview of Spain’s national cybersecurity system

As a member state of the European Union (EU), the Spanish national cybersecurity system was strongly impacted by European policies. EU policy to combat cybercrime originated with the Budapest Convention [12], promoted by the Stockholm Programme [21]. This programme defined EU priorities for the development of an area of freedom, security and justice (2010–2014). It indicated that EU member states should, as soon as possible, “ratify the 2001 Council of Europe Convention on Cybercrime” as a “legal framework of reference for fighting cybercrime at global level” (p. 22). In parallel to the Stockholm Programme, the EU Internal Security Strategy [13] included, for the first time, objective 3 exclusively dedicated to proposing measures to improve the security levels of citizens and businesses in cyberspace.

Subsequent legal instruments adopted by the EU have influenced the current institutional landscape of cybersecurity in Spain. In the legal sphere, the approval of the Directive on security of network and information systems (NIS Directive) heavily impacted the Spanish policies.^1^ The Directive draws a governance model based on critical infrastructure protection and operators of essential services through a multi-stakeholder and multilevel approach, sustained by a strong emphasis on cooperation and information exchange among member States [2]. In addition, the EU created in 2004 the European Union Agency for Cybersecurity (ENISA) to facilitate this cooperation and to help harmonise cybersecurity policies and practices in the Member States, which will be complemented by the work of the European Cybersecurity Competence Network and Centre (ECCC). EU instruments and agencies have shaped Spanish cybersecurity policies in recent years. Their influence can be seen in the designation of competent authorities, the creation of national Computer Emergency Response Teams (CERTs) and the distribution of competencies according to whether the protection is oriented towards public or private networks and critical infrastructures.

**Fig. 1:**
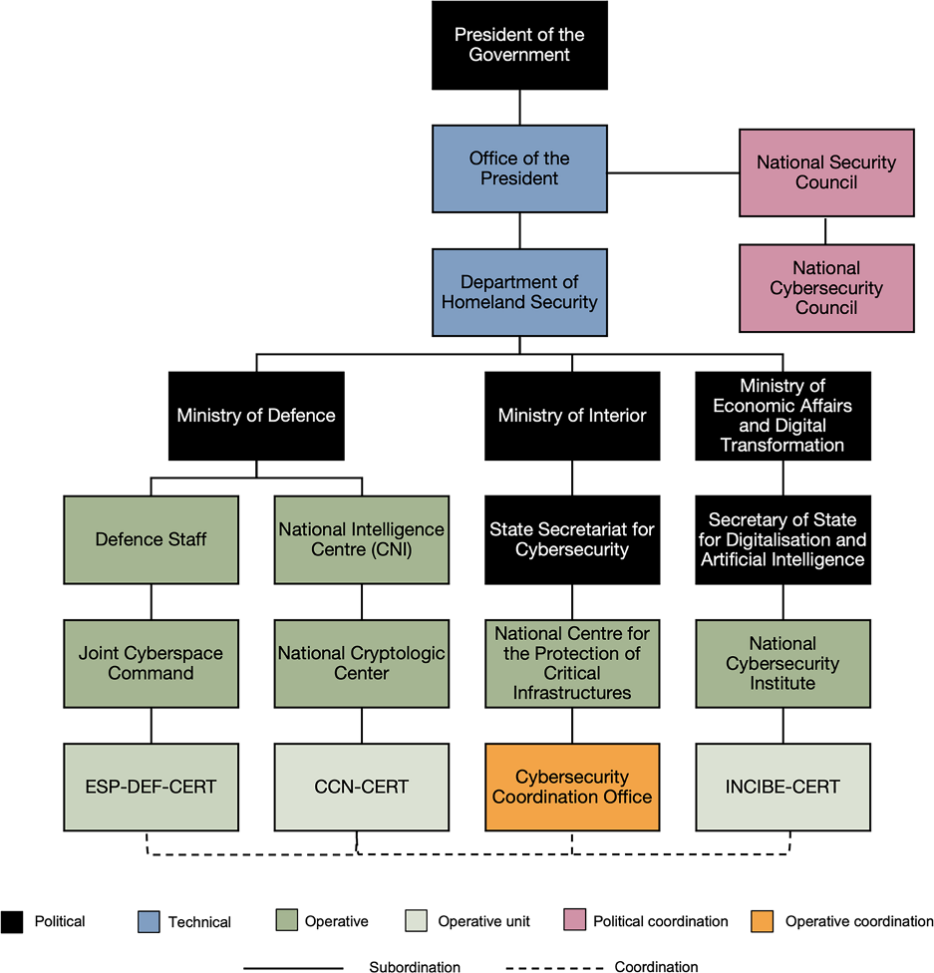
National cybersecurity system according to Royal Decree 311/2022, of 3 May, regulating the National Security framework. Notes: There are three types of government bodies in Spain, political, technical, and operative (see footnote 2). Abbreviations refer to the Joint Cyberspace Command CERT (ESP-DEF-CERT), the CERT of the National Cryptologic Centre (CCN-CERT), and the CERT of the Spanish National Institute of Cybersecurity (INCIBE-CERT).

A simplified structure of the national cybersecurity scheme can be seen in Fig. 1. In accordance with Article 97 of the Spanish Constitution,^2^ the Presidency of the Government is the starting point for all executive institutions in Spain. Administratively dependent on the Presidency of the Government is the Cabinet of the Presidency of the Government (Cabinet Office), the aim of which is to advise the Prime Minister of Spain on matters of interest and, specifically, as stated in Article 2.1 e) of Royal Decree 136/2020, of 27 January, which restructures the Presidency of the Government, ‘in matters of National Security’. The Department of Homeland Security (hereinafter, DSN) reports to the Cabinet Office. The DSN was created by Royal Decree 1119/2012 of 20 July, by which the structure of the President’s office of the Government is modified, and currently regulated by Royal Decree 136/2020 of 27 January. The DSN is a technical assistance body on national security affairs at the service of the Prime Minister of Spain.

The DSN is structured in seven offices, including the Cybersecurity and Disinformation Office.^3^ This office coordinates the different ministries and cybersecurity institutions at a national level. The DSN holds the vice-presidency and the secretariat of the National Cybersecurity Council (hereinafter, CNC). Indeed, the CNC is the main cybersecurity policy coordination institution in Spain. It was formed by Agreement from the National Security Council on 5 December 2013 and formally constituted on 24 February 2014. The CNC is a collegial body supporting the National Security Council with the mission of strengthening the coordination, collaboration and cooperation relations between the different Public Administrations with competences in cybersecurity matters (according to article 21 of Law 36/2015, of 28 September, on National Security). Since the beginning, the CNC has been in charge of leading the development of national cybersecurity strategies (2013 and 2019).

Dependent on the Presidency of the Government and coordinated by the DSN and the CNC, three Ministries—i.e., Ministry of Defence, Ministry of Interior, and Ministry of Economic Affairs and Digital Transformation—host the four leading cybersecurity operational bodies.^4^ According to Spanish legislation (Article 33 of Royal Decree 311/2022, regulating the National Security framework) these are: the Joint Cyberspace Command (henceforth, MCCE), the National Cryptologic Centre (henceforth, CCN), the National Centre for the Protection of Critical Infrastructure (henceforth, CNPIC), and the Spanish National Cybersecurity Institute (henceforth, INCIBE).^5^ These three bodies have divided up the functions of detecting and responding to cybersecurity incidents within Spain’s borders.

### Defence of national security

The Ministry of Defence includes two cybersecurity operational bodies: the MCCE and the CCN. On the one hand, the MCCE is ‘the body responsible for the direction, control and execution of actions to ensure the Armed Forces’ freedom of action in the cyberspace domain’, as well as for responding to cyber threats to national security from abroad (Article 13.1 of Royal Decree 521/2020 of 19 May, which establishes the basic organisation of the Armed Forces). The MCCE hosts the ESPDEF-CERT, the Ministry of Defence’s Computer Emergency Response Team. According to Article 33.4 of Royal Decree 311/2022, it must receive notifications of all cybersecurity incidents that may impact the Ministry of Defence’s functioning or the Armed Forces’ operability.

On the other hand, the CCN is part of the National Intelligence Centre (henceforth, CNI), the Spanish intelligence service. Its functions and structure are regulated by Royal Decree 421/2004 of 12 March 2004, based on Law 11/2002 of 6 May 2002. The Royal Decree entrusted the CNI with the duties related to the security of information technologies (Article 4.e) and the protection of classified information (Article 4.f). Since then, the CCN has played a significant role in developing cybersecurity in Spain. The CCN contributes by (i) increasing cybersecurity knowledge through the CCN-STIC guides^999^, aimed at both public administrations and private companies, (ii) detecting, through SAT INET^992^ probes, cyber-attacks affecting public administrations’ networks and systems, (iii) responding to and mitigating cyber-attacks affecting public administrations’ networks and systems, which the CCN carries out through the CCN-CERT, (iv) the certification of information technology products and systems, cryptologic products, and equipment, systems and facilities that handle information classified as “Confidential” or higher, all through its Certification Body, and (v) the dissemination of cybersecurity culture.

The basic structure of the CCN is formed by a Technical Office and a Secretariat, the Cybersecurity Department, the Information and communication technology (ICT) Security Products and Technologies Department and the Certification Unit. The Cybersecurity Department comprises the Technical Office, the Communication Department, and three departments dedicated to prevention, detection and/or response. In the area of prevention, the CCN hosts the cybersecurity regulations and services department, and in the area of detection and response, the CCN-CERT and the Cybersecurity Operations Centre (SOC).

The CCN-CERT—i.e., the CCN computer emergency response team—was created in 2006 as a national CERT.^6^ Its services are described in Chapter IV, article 34.1 of Royal Decree 311/2022, and include cyber incident response, research and dissemination of cybersecurity best practices and information on vulnerabilities and threats.^7^ The Early Warning Systems (henceforth, SAT), developed by the CCN in 2008 to detect incidents affecting public administrations, play an essential role in CCN-CERT missions. These systems operate by means of probes: high-performance servers that monitor and manage Internet traffic. SAT is included in the SOC, which are responsible for monitoring and detecting threats in the daily operations of the public administrations’ information and communications systems.

The ‘Cybersecurity Shock Plan’, approved on 25 May 2021, further increased the CCN’s missions. This Plan was developed with the aim of reinforcing cybersecurity capabilities in Spain, partly as a reaction to the increase in cybercrime suffered during the Covid-19 pandemic [7] and the two cyber-attacks that affected the Spanish Ministry of Labour and the State Employment Service in March and June 2021. The Shock Plan includes the creation of the Centre of cyber security Operations of the General state administration and its Agencies (hereinafter COCS). The COCS will ‘reinforce the capacities for surveillance, prevention, protection, detection, response to cybersecurity incidents, advice and support for cybersecurity management in a centralised manner’. The COCS, which is expected to be founded within two years, will be operated by the CCN and managed by the Secretariat for Digital Administration of the Secretary of State for Digitalisation and Artificial Intelligence, in a further step that strengthens the intelligence service’s position as a central cybersecurity player in Spain.

### Protection of critical infrastructures

One area of particular concern for States is the protection of their critical infrastructures, defined as the physical and information technology facilities, networks, systems and equipment which are essential for the maintenance of vital societal functions. These infrastructures support Spain’s essential services, including communications services, water and waste management, and energy and nuclear facilities. A cyber incident affecting any of these infrastructures would have a very high disruptive impact. Due to their need for special protection, both the EU, through Directive 2008/114/EC, and Spain, with the Law 8/2011, 28 April, by which establish measures for the protection of critical infrastructures, the so-called ‘PIC Law’, have developed regulations and created bodies to protect them.

CNPIC is the critical infrastructure protection body created in Spain, which was established by the PIC Law. The CNPIC reports directly to the Secretary of State for Security, within the Ministry of Interior. The CNPIC aims to promote, supervise and coordinate all policies and activities related to the protection of critical infrastructures in Spain. To this end, it includes the Cybersecurity Coordination Office (OCC)—formerly the Cybernetic Coordination Office—which coordinates INCIBE-CERT (see Sect. 2.3) and the CCN-CERT responses.

### Promotion of digital society

INCIBE is the evolution of the former National Institute of Communication Technologies (INTECO). INTECO was created in 2006 as an instrument of the Secretary of State for Communication and Information Society, then under the Ministry of Industry, as part of the ‘*Plan Avanza*’. The objective of the *Plan Avanza *was to promote the use of ICTs by citizens and companies. At that time, the decision was taken to create a new Institute with the aim of implementing the *Plan Avanza* measures; in other words, the Institute was not intended to focus on developing new policies but to ensure that the policies—and their measures—end up reaching citizens and companies.

While at the beginning INTECO had three pillars—software quality, web accessibility, and cybersecurity—in 2013 it was decided that the Institute would focus exclusively on cybersecurity. As a result, INTECO was renamed INCIBE. Currently, INCIBE is a state-owned company whose main function is to provide public cybersecurity services to citizens, companies, and operators of essential services. As part of INCIBE, the INCIBE-CERT—with the CCN-CERT and ESPDEF-CERT—is one of the three national CERTs and the largest in Spain. It is a service that operates 24/7 and receives incident notifications from citizens, companies, and critical operators (article 33.7 of Royal Decree 311/2022). Its objective is to analyse these incidents, mitigate them and provide solutions so that victims can recover. In addition, INCIBE has recently offered a speed-dial number for cybersecurity incidents, 017. On the 29 September 2022, the INCIBE was designated as the National Coordination Centre of the European Cybersecurity Competence Centre [38]. This designation is based on the fact that INCIBE ‘has extensive experience in the sector and expertise in technology, research and innovation, and has established itself as a benchmark entity for the development of cybersecurity and digital assurance for citizens, academic and research networks, professionals, companies, and particularly for strategic sectors’ (paragraph two of the official press release in Spanish National Cybersecurity Institute [38]).

Looking exclusively at the legislation, Spain has implemented a coherent and clear institutional design, following a scheme similar to the European one of distributed competencies according to different areas of cybersecurity [10]. However, this article argues that the practice of cybersecurity governance rarely respects the order and coherence indicated by the literal formality of legal texts. Having said that, research usually focuses on the ‘underlying (normative) assumptions and claims’, which ‘often take precedence over empirical enquiry’ [33, p. 681]. In contrast, by studying reality, we may find that the fight against changing, complex and hybrid cyber threats, requires the deployment of a great diversity of resources, turning this fight into a day-to-day work of public organisations with private companies.

## Methodology

Data were collected through a Delphi study and 34 in-depth interviews with security managers and cybersecurity experts from July 2020 to February 2021. This research design allowed us to (i) obtain a list of public and private nodes with cybersecurity competencies in Spain, (ii) understand what resources these nodes possess and exchange, and (iii) understand their collaborative relationships. The study began by conducting a Delphi with cybersecurity experts in Spain.

The Delphi method seeks to obtain a consensus opinion among a sample of experts [14] through a structured and iterative process. It consists of several rounds in which experts express their views anonymously. We chose this method based on previous literature claims that the consensus opinion of a high-level selection of key experts can provide more reliable conclusions than a collection of individual opinions [31].

### Delphi design and conceptual framework

The Delphi study is composed of three rounds. Data for each round were collected through questionnaires hosted on the SurveyMonkey software company server and implemented online. Round 1 (R1) was introductory, so it was comprised of an open-ended question. First, experts were asked to identify the five most relevant nodes of the Spanish cybersecurity system. The link to the R1 questionnaire was sent on 1 July 2020. The R1 question yielded a list of 75 nodes (see Sect. 4). Based on this list, we created five clusters, comprising (i) public organisations (INCIBE, CCN and CNPIC), (ii) law enforcement agencies (i.e., specialised cybercrime units), (iii) army (i.e., Joint Cyberspace Command), (iv) big technology companies, and (v) small and medium-sized enterprises (SME). Then, round 2 (R2) explored capital allocation across these five clusters of stakeholders.

R2 questions were based on Dupont’s [19, p. 85] conceptualisation of five forms of capital. First, economic capital comprises the financial resources available to the node to produce the desired result. In this paper, we work with a twofold dimension of economic capital: On the one hand, direct economic capital, incorporated into the organisation, which we define as the organisational budget. On the other hand, indirect economic capital is both the employees and the quantity and quality of the organisation’s infrastructure and technology. Second, political capital is defined by the node’s capability to influence decision-making in security policy and align it with its interests. Thus, political capital measures the node’s proximity to the government machinery. Third, cultural capital is defined as the accumulated knowledge and experience of the node regarding security provision. Forth, the node also needs symbolic capital to act, represented by the mechanisms that confer legitimacy on a node when acting on a given security problem, as well as its power to speak authoritatively to other nodes. Finally, social capital is ‘the whole set of social relations that allow the constitution, maintenance and expansion of social networks’ [19, p. 86].

Based on this conceptualisation, we defined six statements assessing four types of capital (i.e., economic, political, cultural and symbolic) ([19, 36]; see Table 1). Note that we decided not to include social capital in our analysis. The reason can be found in this capital’s description: ‘[*social capital*] depends on the size of the network of connections that [*the node*] can effectively mobilise and on the volume of the capital (economic, cultural or symbolic) possessed in his own right by each of those to whom [*the node*] is connected’ [5, p. 21]. This goal required a different method than that of the Delphi study, so we decided to investigate social capital through social network analysis techniques and in-depth interviews (see Sect. 3.3).

**Table 1:** Type of capital, definition, and question for the Delphi study

Capital	Definition	Question
Economic	The budget available to the node, but also what the budget makes possible: human resources, infrastructure, etc.	[*The following organisations* …] are adequately staffed to carry out their functions in the field of cybersecurity in Spain (*Personnel*)
		[*The following organisations* …] have the necessary infrastructure and technology to carry out their functions in the field of cybersecurity in Spain (*Infrastructure & technology*)
		[*The following organisations* …] have the necessary funding to carry out their functions in the field of cybersecurity in Spain (*Funding*)
Political	Node’s ability to influence policy decisions on cybersecurity	[*The following organisations* …] can influence the government’s cybersecurity agenda
Cultural	Node knowledge on cybersecurity	Its staff have excellent technical cybersecurity expertise
Symbolic	Legitimacy of the node	Citizens and businesses should follow their cybersecurity advice, even if they do not always agree with it

The link to the R2 questionnaire was sent on 21 September 2020. Over three weeks, weekly reminders were sent before the questionnaire was closed. After the interim analysis of the R2 responses, the second round (R2) questionnaire was accompanied by graphs showing the experts’ answers to R1. Since the purpose of Delphi studies is to measure consensus among experts, questions on which agreement was not reached in R2 are asked again in R3.

### The expert panel

Experts were defined as individuals with power as well as and political and operational decision-making capacities, occupying senior positions in their organisations, with deep knowledge of organisations within the cybersecurity ecosystem. The experts invited to participate were obtained by identifying those specific individuals with the greatest expertise within the five most relevant categories of organisations and groups of organisations (see Table 2).

**Table 2:** List of relevant organisations and group of organisations to get participants

Cluster	Organisations or groups of organisations
Public sector	National Cryptologic Centre (CCN)
	National Centre for the Protection of Critical Infrastructures (CNPIC)
	National Cybersecurity Council (CNC)
	National Security Council
	Department of National Security (DSN)
	Coordinating Prosecutor’s Office for Computer Crime
	Spanish National Cybersecurity Institute (INCIBE)
Security institutions	Ertzaintza
	Technological Investigation Unit of the National Police
	Telematic Crimes Group of the Civil Guard
	Joint Cyber Defence Command
	Mossos d’Esquadra
Tech companies	Technological consultancy firms
	Technology-based companies
	Internet service provider companies
	Telecommunications companies
	Cybersecurity service companies
Non-tech companies	Private security services companies
	Other companies (banking, transport, energy, etc.)
Academia	Universities
	Research institutes

Among the 275 cybersecurity experts who were invited, 129 responded to the Round 1 questionnaire (46.9% response rate). Of these responses, 104 were from men (80.6%) and 25 from women (19.4%). While males are over-represented in our sample, the proportion of women is higher than the existing population of women experts in cybersecurity in Europe, which stands at 7% [35]. The gender proportion was maintained through R2 and R3. Table 3 summarises the socio-demographic and occupational distribution of the panel of experts.

**Table 3:** Socio-demographic and occupational distribution of the Delphi expert panel

		Round 1 (*N* = 129)		Round 2 (*N* = 110)		Round 3 (*N* = 104)	
		*n*	%	*n*	%	*n*	%
Sex							
	Male	104	80.6	90	81.8	84	80.8
	Female	25	19.4	20	18.2	20	19.2
Age							
	25 or less	3	2.3	2	1.8	2	1.9
	26-35	18	14	13	11.8	13	12.5
	36-45	51	39.5	45	40.9	43	41.3
	46-55	42	32.6	35	31.8	33	31.7
	56 or more	15	11.6	15	13.6	13	12.5
Education							
	High school graduate/diploma or equivalent	4	3.1	3	2.7	3	2.9
	Trade/technical/vocational training	10	7.8	8	7.3	6	5.8
	Bachelor’s degree	27	20.9	25	22.7	24	23.1
	Master’s degree	61	47.3	51	46.4	50	48.1
	Doctorate degree	27	20.9	23	20.9	21	20.2
Sector							
	Academia	20	15.5	16	14.5	16	15.4
	Non-tech companies	39	30.2	30	27.2	29	27.9
	Public sector	20	15.5	19	17.3	18	17.3
	Security institution	24	18.6	21	19.1	20	19.2
	Tech companies	26	20.2	24	21.8	21	20.2
Years of experience							
	Less than 5 years	38	29.5	32	29.1	31	29.8
	6–10 years	32	24.8	30	27.3	27	26.0
	11–20 years	46	35.7	36	32.7	35	33.7
	More than 20 years	13	10.1	12	10.9	11	10.6

### Post-Delphi interviews

To obtain the data to measure social capital, R2 participants were specifically asked about their availability for a post-Delphi interview. Of the experts participating in the Delphi study, 34 agreed to be interviewed. We interviewed experts from the public sector (*n* = 6), police and the military (*n* = 8), technology company executives (*n* = 11), CISOs (*n* = 8), and one academic. The interviews were conducted between 17 December 2020 and 5 February 2021, with an average duration of 48 min. All interviews were conducted by video call or telephone call, at the interviewee’s preference. In the interviews, the expert was asked about the collaborative relationships between the nodes who had appeared in the Delphi study to organise the cybersecurity collaboration networks from which social capital could be obtained. Additionally, experts were asked to elaborate on the questions in R2 where no consensus was reached.

### Data analysis

The experts’ responses to R1 resulted in a list of 75 actors (see Annex 1). The actors were then coded based on the type of organisation (i.e., company, government, etc.) and territorial scope. The coding was performed individually by the two authors based on a desk search, and the inter-rater reliability was calculated using Cohen’s kappa [11]. As a result, a good inter-rater agreement was obtained (*κ* = 0.71).

To identify which of them were considered the most relevant, we assumed that if a node had been mentioned by a greater number of experts and ranked higher among the five options requested, there would be a greater consensus on the greater relevance of that specific stakeholder for cybersecurity in Spain. Thus, we calculated the weight for each of the 75 nodes. To obtain the weight, we compute the number of times a node was mentioned in a specific position (1st, 2nd, …) multiplied by five when it was mentioned in the first place, by four when it was mentioned in the second place, by three when in the third place, by two when in the fourth place, and by one when in the fifth place. This calculation yielded a number for each of the nodes, which ranged from 1 if it had been mentioned in the fifth position by only one of the experts to 645 if the entire panel of experts had mentioned it as the most relevant cybersecurity node in Spain in the first position of the five hierarchy options (i.e., the result of calculating 129 mentions in the first position or, in other words, 129 × 5 = 645). The result was then normalised by dividing the actor’s weight by the maximum weight (i.e., 645).

We used the interquartile range (IQR) to assess the consensus. The consensus is reached for scales with five response levels when IQR is ≤ 1, so 50% of the responses are within one point away on the scale [37]. The questions on which the experts did not reach consensus in R2 were asked again in R3, including the feedback from R2. Then, R3 responses were analysed by calculating the changes in the responses to check if the feedback provided in R2 led to a greater convergence among the experts. Changes in the means (*M*) and standard deviations (*SD*) of the responses to the questions included in R2 and R3 were calculated by computing a simple index with the *M* and *SD* multiplied by 100 (see Eq. 1). The descriptive analyses of participants’ responses in R2 and R3 (i.e., mean, standard deviations, and IQR) were performed with SPSS version 28.0.

1 $${\%}\,\text{of change}=\left(1-\frac{\overline{\mathrm{x}}/\sigma _{R2}}{\overline{\mathrm{x}}/\sigma _{R3}}\right)\times 100$$

The interview content that enriched the Delphi study’s results was coded with NVivo. The following codes are used to present the quotations from the experts: PS (public sector), PFA (security institutions), TC (tech companies), OC (private companies), and A (academia), as well as male (M) or female (F). Finally, this article draws on the interviews to perform the social network analysis (SNA). The decision to perform SNA to understand the social capital and power relations among public and private cybersecurity organisations in Spain was inspired by previous studies on security networks (e.g., [20, 33]). SNA view organisations as a ‘social or relational structure that can be unpacked by various mathematical and analytical techniques’ [33, p. 683]. Furthermore, SNA assumes that actors situated in central positions with governance networks have a greater ability to influence the course of events [20, 33].

The present study uses the concept of brokerage to analyse the distribution of social capital in the network. The literature defines broker agencies or organisations as those that hold key structural positions in networks, thus accumulating more influence and power to control the diffusion of other network capitals [30]. We measured the extent to which cybersecurity organisations are positioned as brokers using the betweenness centrality [22]. Betweenness centrality measures ‘how often a given node falls along the shortest path between two other nodes’ [4, p. 174]. Nodes positioned between other nodes are assumed to have a greater ability to influence information and resource exchange flows.

## Results

### Results of round 1

**Fig. 2:**
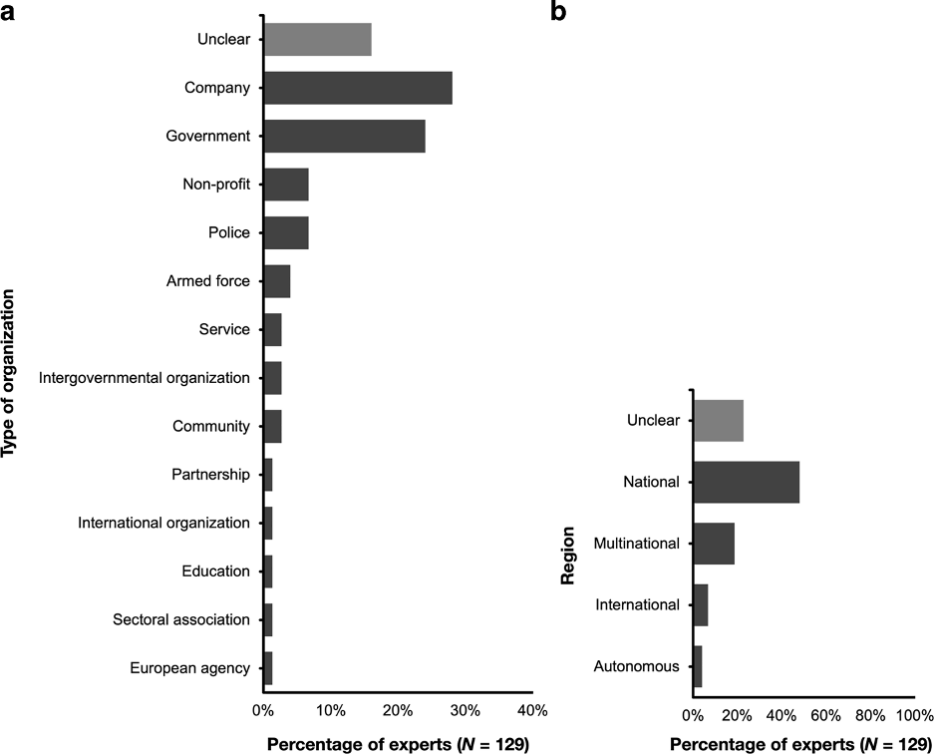
Characteristics of the 75 stakeholders mentioned by the panel of experts during round 1 by **a** type of organisation, and **b** region

The 129 Delphi experts identified 75 nodes within the national cybersecurity system (the full list can be found in Table 6 in the Appendix). 92.2% of the experts (*n* = 119) included the INCIBE as one of the most relevant nodes in the Spanish cybersecurity landscape, followed by the CCN (*n* = 114, 88.4%), the CNPIC (*n* = 50; 38.8%), the DSN (*n* = 49; 38.0%), and the Joint Cyberspace Command (MCCE) (*n* = 45; 34.9%). The list includes 70.7% (*n* = 53) specific names of organisations and companies (e.g., CCN) and 22 (29.3%) unspecific groups (e.g., ‘universities’, on ‘public institutions’). Fig. 2 shows the characteristics of the 75 nodes mentioned by the panel of experts based on the type of organisation and territorial scope. The experts mostly mentioned companies (*n* = 21; 28%)—including company-related clusters such as ‘cybersecurity companies’—and public bodies (*n* = 19; 24%). In Fig. 2, public bodies have been differentiated from the police and Army—mentioned in Sect. 1 as the traditional security bodies—for comparative purposes. However, they are all part of Spain’s public administration (see another example of this approach in [27]).

Based on the territorial scope, most of the nodes mentioned by the panel of experts operate at a national level (*n* = 36, 48%). In this category, we included public organisations with national competencies, private companies offering their services only in Spain, and clusters such as the hackers’ community. The experts also included multinational, international, and autonomous nodes.

**Fig. 3:**
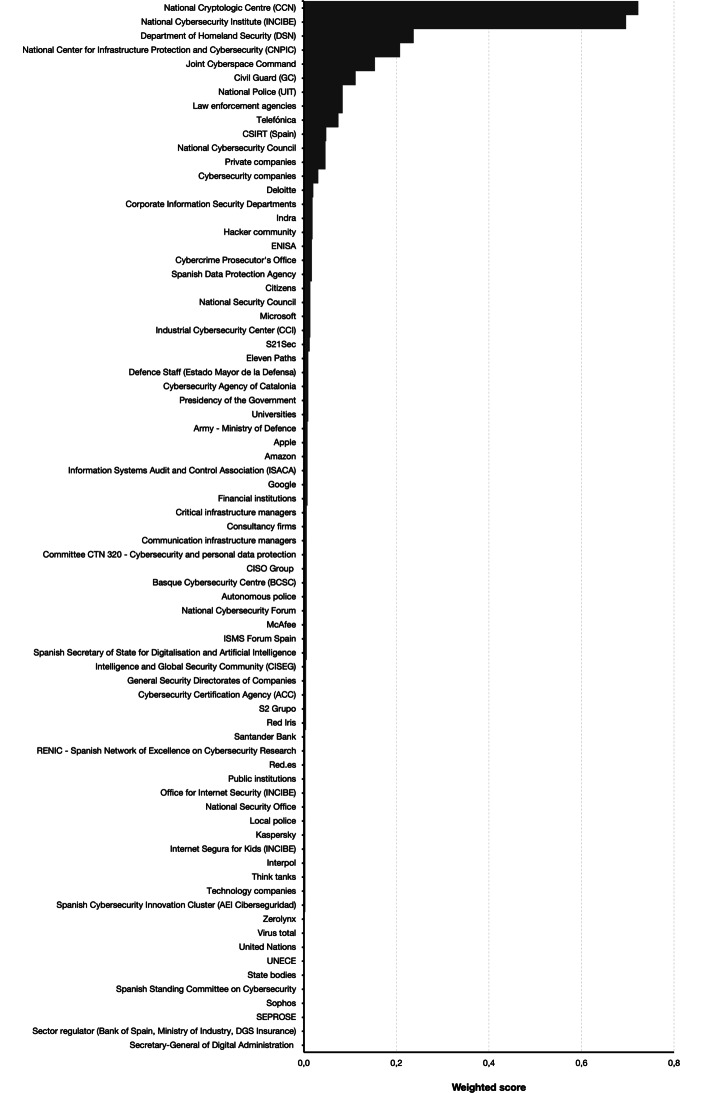
Weighted scores for the 75 actors identified by the panel of experts in Round 1

The results of the weights (see Sect. 3.4) based on the number of mentions and the position in the scale of first to fifth are displayed in Fig. 3. According to Fig. 3, the most relevant node in the Spanish cybersecurity landscape is the CCN, closely followed by the INCIBE. The weighted score calculation reversed the order of the first two positions. This result is because the CCN, when included in the list of nodes, was mentioned in higher positions than the INCIBE—i.e., 51 (39.5%) experts filled in the first position with the CCN, while 40 (31%) did so with the INCIBE. The rest of the identified nodes had significantly lower weighted scores than these two organisations.

### Consensus analysis of R2 and R3

The results of IQR in R2 and R3 are shown in Table 4. The consensus was reached in 19 out of 30 questions (63.3%) (see IQR in brackets) in R2. Respondents showed higher consensus opinions for the questions about large technology companies (83.3%), SMEs (83.3%), and LEAS (66.7%). The least number of consensus questions was about the Army’s capitals (33.3%). Full consensus was reached for all questions on political capital. The 11 questions where consensus was not reached were re-assessed by the panel of experts in R3.

**Table 4:** Descriptive statistics for the five forms of capital

		Round 2 (*n* = 110)			Round 3 (*n* = 104)			*M *change	*SD *change
Capital		IQR	*M*	*SD*	IQR	*M*	*SD*		
Economic (*personnel*)									
	Public organisations	2	3.34	1.09	2	3.11	0.93	−7.40	−17.20
	Law enforcement agencies	2	2.87	1.23	(0)	2.32	0.77	−23.71	−59.74
	Army	2	3.05	1.10	2	2.92	0.88	−4.45	−25.00
	Large tech companies	(1)	4.12	0.84					
	SME	(1)	1.73	0.78					
Economic (*infrastructure & technology*)									
	Public organisations	1.25	3.51	1.07	(1)	3.51	0.85	0	−25.88
	Law enforcement agencies	2	3.00	1.15	(1)	2.57	0.94	−16.73	−22.34
	Army	2	3.16	1.06	(0)	2.99	0.69	−5.69	−53.62
	Large tech companies	(1)	4.43	0.76					
	SME	(1)	1.72	0.79					
Economic (*funding*)									
	Public organisations	2	3.10	1.13	2	3.29	1.00	5.78	−13.00
	Law enforcement agencies	(1)	2.45	1.05					
	Army	2	2.70	1.10	(1)	2.79	0.72	3.23	−52.78
	Large tech companies	(1)	4.45	0.69					
	SME	(1)	1.65	0.78					
Political									
	Public organisations	(1)	4.14	0.77					
	Law enforcement agencies	(1)	3.33	0.98					
	Army	(1)	3.39	0.97					
	Large tech companies	(1)	3.70	0.93					
	SME	(1)	1.64	0.76					
Cultural									
	Public organisations	(1)	4.26	0.81					
	Law enforcement agencies	(1)	4.12	0.89					
	Army	2	3.91	1.02	(0)	3.74	0.76	−4.55	−34.21
	Large tech companies	(1)	4.61	0.56					
	SME	(1)	1.92	0.84					
Symbolic									
	Public organisations	(1)	4.39	0.68					
	Law enforcement agencies	(1)	4.47	0.73					
	Army	(1)	4.05	1.05					
	Large tech companies	1.25	3.29	1.04	(1)	2.90	0.77	−13.45	−35.06
	SME	1.25	2.84	1.09	(1)	2.69	0.65	−5.58	−67.69

Brackets mark questions where final consensus was reached, *i*.*e*., an interquartile range ≤ 1

*SME* small and medium-sized enterprises

The responses of the experts in R3 showed greater consensus. Experts achieved consensus over the economic capital of public organisations (*infrastructure & technology*), LEAS (*personnel*, and *infrastructure & technology*), and the Army (*infrastructure & technology, *and *funding*). Consensus on the economic capital (*personnel*) was neither reached for public organisations and the Army, nor the assessment of the *funding* received by the public organisations. The variations in means and standard deviations between R2 and R3—expressed in percentages—are shown in Table 4. All standard deviations were considerably reduced. This suggests that the Delphi method reduced the dispersion of expert responses, even though the consensus was not reached on all the statements. A higher proportion of experts selecting the options ‘disagree’ or ‘strongly disagree’ in R3 led to a reduction in the means for the public organisations’ *personnel* and the *infrastructure & technology*.

Experts disagreed on the cultural capital of the Army. During R3, the experts reached a consensus (IQR = 0) over this question on the ‘agree’ response (70%), yielding a 34.2% decrease in the SD. Moreover, the IQR for the symbolic capital of large technology corporations and SMEs was 1.25. Consequently, these two groups were re-assessed in R3. The feedback obtained on these two questions during R2, rather than aligning the responses towards agreement or disagreement positions, prompted the experts to position themselves in the middle ground of ‘neither agree nor disagree’.

### Descriptive results for economic, political, cultural, and symbolic capital

The results shown in Table 4 are consistent for all three forms of economic capital (i.e., *personnel, infrastructure & technology*, and *funding*) across the five groups of actors. Large technology corporations are the best equipped in terms of human resources, *infrastructure & technology*, and *funding*. In contrast, SMEs are the worst equipped in Spain regarding cybersecurity in all three types of economic capital. For instance, 90% of the experts in R2 ‘disagree’ or ‘strongly disagree’ that SMEs have the necessary funding to carry out their functions in the field of cybersecurity. While experts are clear about the economic capital of large technology corporations and SMEs, the same conclusion is not reached for public organisations, LEAS, and the Army. The results indicate that public organisations have slightly more economic capital than the LEAS and the Army. The differences will be explored in more depth in Sect. 4.4 below.

Concerning political capital, experts responded that public organisations have the highest ability to influence the government’s cybersecurity agenda (86.4% positioned themselves between ‘agree’ and ‘completely agree’). In descending order, we can find the large technology corporations, the Army, and the LEAS. In contrast, experts’ opinions on the political capital of SMEs revealed higher consensus, with 84.5% of experts assessing that they had insufficient capacity to influence the government’s cybersecurity agenda.

This same pattern is repeated in cultural capital. Public organisations, LEAS, the Army, and large technology companies’ personnel is assessed by experts as having excellent technical cybersecurity expertise. However, Table 4 reveals that symbolic capital is placed in public bodies. A total of 92.7% of the experts in R2 considered that citizens should follow the recommendations from public organisations, 91.8% from the LEAS, and 76.4% from the Army’s Joint Cyberspace Command. In contrast, experts’ responses on the symbolic capital of large technology corporations and SMEs are ambiguous, as the most frequent option in R3 was ‘neither agree nor disagree’.

### Qualitative analysis of post-Delphi interviews

The qualitative analysis of the expert’s interviews offered the following conclusions.

#### Public organisations (INCIBE, CCN, and CNPIC)

Interviewees agreed there is an unbalanced distribution of skilful and specialised personnel among INCIBE, CNN and CNPIC. These three organisations lack sufficient capacity to recruit highly skilled employees. However, INCIBE and CCN need qualified employees more urgently than CNPIC. There are two main reasons for CCN and INCIBE’s staffing shortages. First, budget constraints: ‘[*Their*] funding […] comes from public funds, so that, depending on the moment, there may be difficulties when launching calls for personnel recruitment’ (F-PS3). Staffing is to a lesser extent also affected by these agencies’ capacity to retain their employees. Many employees, once trained, move on to another body within the Public Sector—or even move from the public to the private sector, where they obtain better working conditions and higher salaries.

Experts agreed that the CCN is the best equipped in terms of infrastructure & technology, followed by the INCIBE. Lastly, the interviewees provided further explanations for the cultural capital of INCIBE, CCN and CNPIC. According to the panel, the CCN members are among the most skilled and knowledgeable in cybersecurity, followed by those at INCIBE; CNPIC would lack cultural capital compared to its two peers. In this sense, the scarcity of highly specialised staff is affecting the CCN more than the INCIBE, because ‘[…] INCIBE […] is focused on ordinary citizens, which requires [to hire] less technical profiles’ (M-TC4).

#### LEAS (specialised cybercrime units)

According to interviewees, the number of officers within the specialised cybercrime units of the National Police and the Guardia Civil is significantly lower than they would need to fight cybercrime effectively. As expressed by a police officer, ‘Cybercrime has been growing steadily. [*However*], the Guardia Civil and the National Police [*have not*] increased their number of employees to the necessary level to fight this rise in cybercrime’ (F-PFA20).

#### Army (joint cyberspace command)

Staffing shortages in the MCCE are due to the youth of this organisation—established in 2020. However, this shortage has been compensated for by various outsourcing processes:

In [*public*] agencies, the planned number of employees is never reached because another new agency emerges with needs, there’s another priority, and so on. In my opinion, the Joint Cyberspace Command is understaffed. What happens, then? Well, it’s compensated with consultancies and support from private companies. For example, with ISDEFE personnel (F-PFA2).

Some of the experts interviewed referred that the armed forces personnel were not adequately trained in cybersecurity. The origin of this problem may be found in the Army’s recruitment practices, which are considered outdated and mismatched with cyber-Spain’s defence needs. Staffing shortages also affect its cultural capital, as the rules that regulate military personnel promotion favour a high turnover in job postings. These regulations make training military personnel in strategic, tactical, and technical cyber-defence skills highly inefficient, as they likely will eventually change their posting.

#### Large technology corporations

Interviewees offered divergent answers on the symbolic capital of large technology corporations. On the one hand, some experts identified ICT companies as the best prepared to provide recommendations and advice to citizens and other enterprises. For example, ‘Large corporations spend most time and resources researching IT security and implementing measures, so citizens should follow their recommendations’ (F-OC12). However, cybersecurity outsourcing is negatively perceived by some experts. For instance, one police officer stated that he was ‘against this privatisation of national security tasks. This transfer of obligations is a structural danger that unbalances Western democracies’ (M-PFA2). For this reason, most experts support the idea that public organisations should provide cybersecurity recommendations, not private companies, as these organisations’ goals are society’s general interest and not private economic profit.

#### SMEs

Finally, SMEs are perceived as lacking all four types of capital. Interviewees repeatedly affirmed that the SMEs are ‘clueless about IT security measures’ (M-OC21). However, their low symbolic capital is not due to a lack of knowledge but because public organisations are the only ones entitled to do so: ‘[…] I believe that the obligation of compliance that we citizens have is only enforceable through public mandates from public administrations, not from private companies, however well-intentioned they might be’ (M-TC5).

### Social network analysis

**Fig. 4:**
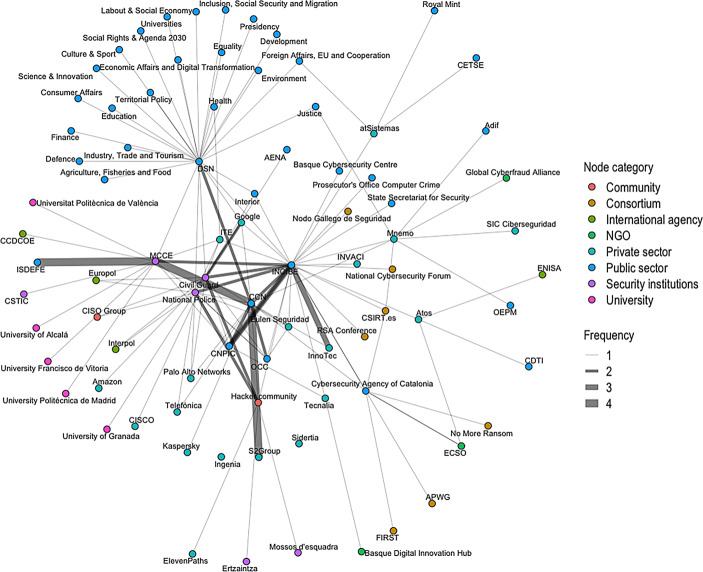
Network of links between relevant cybersecurity agents within Spain

Social capital was analysed through social network analysis techniques. The 34 experts interviewed reported a total of 133 collaborative relationships between 84 cybersecurity actors. Fig. 4 represents the network of these collaborative relations. In the figure, the nodes represent the agents, and each line connecting the nodes represents a collaborative tie. Thicker lines represent the links that were identified by a greater number of experts. As can be observed, these thicker lines link the INCIBE with InnoTec—a Spanish cybersecurity services company—the CCN, the National Police and the Guardia Civil.

A close examination of Fig. 1 reveals a close relationship between the INCIBE and the CCN, consistent with the structure of the national cybersecurity scheme in Spain (see Sect. 2 above). INCIBE’s public is both private companies and citizens, which is why it appears connected to almost all the companies shown in Fig. 4. On the other hand, CCN’s audience consists exclusively of public bodies. However, the reality is more intricate. According to the experts interviewed, INCIBE and CCN compete for the same niche: the management of cyber-incidents against the state’s critical infrastructures. Coherently with this complex situation, Fig. 4 shows how both organisations are reportedly strongly linked to the CNPIC, located very close to both INCIBE and CCN, at the network’s core.

The distribution of betweenness within the collaborative cybersecurity network is shown in Table 5. We have included those 10 nodes with the highest score. As observed, INCIBE holds the most central position in the network, closely followed by the Department of Homeland Security in Spain. The CCN have the third most central position based on betweenness. Betweenness results also reveal the central role of LEAS. Almost all experts mentioned some form of collaboration either with the National Police or the Civil Guard. Finally, we find that the list of the most central organisations includes two technology companies and the hacker community.

**Table 5:** Betweenness centrality scores for collaborative networks in cybersecurity in Spain

Ranking	Node	Betweenness
1	Spanish National Cybersecurity Institute (INCIBE)	1546.97
2	Department of Homeland Security (DSN)	1471.39
3	National Cryptologic Centre (CCN)	630.58
4	Joint Cyberspace Command (MCCE)	562.58
5	Civil Guard	399.78
6	National Police	345.40
7	Mnemo	341.50
8	Cybersecurity Agency of Catalonia	310.35
9	Hacker community	246.50
10	atSistemas	175.18

### Collaboration between nodes

During the interviews, the experts raised two major topics: types and motivations of the collaborations and barriers. The interviewees mentioned that the actors collaborate by exchanging information, developing cyber-attack prevention strategies, and responding jointly and in coordination to cyber-incidents. All these activities depend on the seriousness of the cyber-attack. Only when companies perceive a severe risk to their assets do they agree to share information and collaborate with other companies and public bodies. For example, one interviewee mentioned that the collaboration increased during the WannaCry crisis,^8^ when the CCN worked with Microsoft in the forensic investigation.

In addition, public-private collaboration usually occurs when the cyber incident affects a public institution or when it targets critical infrastructures or essential services. In the former case, the collaboration is established out of necessity because the public institution may require private services to tackle the cyber incident. For example, in March 2021, the Spanish State Public Employment Service suffered a massive ransomware attack, which resulted in the Ministry of Labour urgently awarding contracts with technology and cybersecurity companies [1]. In the latter case, the collaboration between public institutions, critical infrastructure, and essential services operators is compulsory,^9^ which ‘resulted in an increase in trust between these actors’ (M-PS7).

The lack of trust was the barrier to collaboration most frequently mentioned by the interviewees. This prevents actors from sharing sensitive information related to their vulnerabilities. The experts offered three main reasons for the lack of trust. First, there is a perception of disparity between the aims pursued by private enterprises and by public bodies. For example, one expert from the Civil Guard said, ‘private enterprise seeks profit and economic growth, which is inherently opposed to national security principles’ (M-PFA2). Second, public institutions are perceived as inefficient by the experts of the private sector. For example, this was a CISO’s experience:

We recently had an incident, and we started working to solve it. We called Telefónica, and they helped us out. Three days later, INCIBE called us to find out what had happened and to ask us to send them malware samples. There was no response offering support. My perception is that many companies suffer a cyber-attack and public organisations do nothing. (M-OC18)

The third reason for the lack of trust between the public and private organisations can be found in the closer relationship between the public institutions and some selected companies with which they collaborate more often. Outsourcing some of the public organisations’ operations and services to private third parties is one of the consequences of an imbalanced allocation of capitals, especially economic and cultural. In Spain, the three major public bodies in cybersecurity have been linked to specific companies. For instance, respondents reported that the MCCE had outsourced its missions to ISDEFE, a state-owned enterprise. As an example, an expert from a cybersecurity company stated that ‘More than 60–70% of [*the Joint Cyberspace Command’s employees*] came from ISDEFE’ (M-TC6). Another expert mentioned the CCN and INCIBE being linked to two private companies:

The problem with public-private collaboration in Spain is that public bodies are represented explicitly by competing companies. For example, the CCN is known to be S2 Grupo. Since we [*his company*] are competitors, we do not share any critical information with the CCN because it may end up in the hands of the competition. The same happens with INCIBE, which is closely linked to InnoTec. If public bodies are not independent and represent all of us, little can be done [*to increase public-private collaboration*]. (M-TC1)

The second major barrier to collaboration between actors mentioned by interviewees is the lack of cybersecurity awareness and knowledge. The experts interviewed from both the public and private sectors agreed on this point. It is worth differentiating between a lack of information and a lack of awareness here. In the opinion of the experts, there is a reasonable production of public information on cybersecurity in Spain. As such, public administrations, and some large technology corporations’ efforts to make resources available to the public and the business community so that they are informed have been evident. However, these resources have not been sufficient to generate awareness in the public sector:

From the point of view of public organisations, there is awareness. The EU is forcing them to adopt an ‘awareness-raising’ discourse (…) No one can argue that there is a lack of information. But the sad reality is that we see cyber-attacks against companies and citizens daily (…) and, although we are insisted upon repeatedly, we are negligent because cyber-security is a nuisance; it is a cost: keeping us cyber-safe implies more expense and procedures (…) The effort made in communication is reasonable but ineffective, (…) our lack of fear of possible cyber-attacks is unreasonable. (F-TC17)

The problem of lack of cybersecurity awareness is, in their opinion, affecting especially SMEs. According to experts, the problem stems from implementing incorrect awareness-raising strategies by public administrations. These strategies are based excessively on top-down approaches, which have not managed to reach most of the Spanish business community.

INCIBE does not consider that this is a country full of SMEs. Neither citizens nor SMEs have the necessary cybersecurity knowledge to understand the awareness messages, making it very difficult for them to get through. I think the means being used are wrong. I think we need to create solutions designed for SMEs. The CCN has done an excellent job raising awareness among people in management positions. I think INCIBE should do it from the bottom up. (H-TC6)

In addition to these two major barriers to collaboration between actors, interviewees mentioned other minor ones: disparity in working and action protocols; disparity in objectives and strategies; lack of support from organisational leaders; and lack of adequate communication channels, all of which are related to a lack of trust and cybersecurity awareness.

## Discussion and conclusions

The increase in malicious activity in cyberspace has not been matched by a proportionate and effective response from public organisations. For years, scholars have studied adaptations by law enforcement organisations to fight against cybercrime, repeatedly demonstrating that their changes have been late and insufficient [28]. Cybersecurity opened a new niche occupied by new state and non-state actors, whether organisations or communities of individuals. However, scientific studies examining the structure of governance networks in diverse socio-cultural contexts are still needed. The present research examined the cybersecurity governance landscape in Spain by providing an empirical examination of the nodes, their capitals, and collaborative relationships. Our results allow for the following contribution to governance debates.

The results of this study reveal that cybersecurity provision is organised around a multi-stakeholder model, where public organisations are considered the main actors of the system. Specifically, we identified the CCN, the intelligence agency, as Spain’s most relevant actor in cybersecurity. The INCIBE follows the CCN. This state-owned company promotes the prevention of cyber-attacks through activities to raise public awareness and disseminate cybersecurity culture. Even though there is a CERT within INCIBE, its preventive role is more relevant than detecting and responding to cyber-attacks. The study also identified the roles of the MCCE, the army organ in charge of responding to cyber-attacks affecting the military operations of the Spanish Armed Forces; and that of the specialised cybercrime police units of the National Police and the Guardia Civil, with the mission to obtain forensic evidence to prosecute cybercrime suspects.

Spain designed a dual system with two national CERTs, one outside (i.e., INICBE-CERT) and one inside (i.e., CCN-CERT) the intelligence community. Typically, European countries have opted for one or the other [3]. The choice of embedding the national CERT inside or outside the intelligence community has important consequences. In Spain, there is an imbalance in the quantity and quality of information each CERT receives. The CCN-CERT monitor all public-owned networks and systems, thus receiving direct information and data on ongoing cyber-attacks. Moreover, the CCN-CERT can access classified sources. The INCIBE-CERT, in contrast, heavily relies on the information that companies (and the CCN-CERT) voluntarily choose to share. Moreover, information sharing relies heavily on personal relationships of trust. During the course of this research, the authors have observed how the CCN has a more open stance toward society, which contrasts with the opacity that has traditionally characterised intelligence services [15].

Our exploration indicates the possible existence of a superstructural node. Superstructural nodes are defined as formal structures led by network actors to serve converging interests [36, p. 295]. The concept was developed by Burris, Drahos and Shearing, who stated that ‘The superstructural node brings together representatives of different nodal organisations […] to concentrate the members’ resources and technologies for a common purpose’ [8, p. 38]. This is the Spanish National Cybersecurity Forum.^10^ The National Cybersecurity Forum was envisaged as Measure 9, Action Line 4, of the National Cybersecurity Strategy 2019. Finally, it was founded in July 2020. This forum is composed of the DSN (chair and secretariat), INCIBE (first vice-chair) and the CCN (second vice-chair). According to our findings, the three most relevant cybersecurity bodies have joined forces to create a superstructural node to serve as a facilitating channel for public-private collaboration. Specifically, the objectives of the National Cybersecurity Forum are (i) to increase the culture of cybersecurity, (ii) to increase public-private collaboration in industry and R&D, (iii) to promote cybersecurity training in line with market demands, (iv) to orient industry and research towards cyber defence, and (v) to systematise public-private collaboration in regulatory matters. All these objectives match the description of superstructural nodes [8]. Future research should explore the role of the National Cybersecurity Forum in depth.

The analysis of the five forms of capital that the actors possess suggests that Spanish public cybersecurity organisations hold a central position in governance networks, despite the greater economic and cultural capital owned by large technology corporations. SMEs, however, lack the appropriate capitals to be considered relevant actors in cybersecurity governance networks. This may be problematic. In Spain, 99.8% of companies are SME [16]. The finding that SMEs are inadequately prepared to tackle cybersecurity threats is not new and has already been highlighted by numerous research studies [24, 25, 32]. Our study adds new evidence from Spain that supports the need to focus efforts on improving SMEs’ capabilities.

Comparing these results with previous studies in other contexts shows that the Spanish cybersecurity governance model differs from the nodal governance model followed by Anglo-Saxon countries. These countries have been at the centre of most governance studies. According to these studies, countries have adopted a neoliberal discourse [29, 39], which supports the reduction of state intervention and structuring security provision around market laws—i.e., competition, entrepreneurship and contracting out services. For example, one study [40] compared, through congruence analysis, the protection of critical infrastructures against cyber-attacks in the UK and France and found that governance in the UK relied on horizontal coordination of multiple actors.

Our results suggest that Spain has not embraced the neoliberal discourse to the same extent as Anglo-Saxon countries. Spanish public organisations hold a central position in cybersecurity governance networks through political, symbolic, and social capital. This finding provides new empirical evidence of the salient role of anchored pluralism in explaining governance structures. A similar conclusion was obtained in France [36, 40] and Norway [33].

There are two nuances to the above conclusion. First, Spain’s political organisation is highly fragmented. Coherently, this paper identified both national-level and autonomous-level nodes. The two traditionally more pro-independence autonomous communities, the Basque Country and Catalonia, have their own cybersecurity agencies, the Basque Cybersecurity Centre, and the Cybersecurity Agency of Catalonia. And these efforts to create new community-level institutions will likely increase.^11^ The second nuance concerns the reliance of state nodes on third parties. In principle, this solution seems efficient from the capital exchange perspective [41], particularly in cybersecurity where public organisations often lack funding and staffing, while private actors possess economic and cultural capital. As a result, governments delegate the implementation of certain policies to companies [6, p. 4], also in Spain. However, trust relations between the government and selected companies may diminish other stakeholders’ trust in public institutions. As Madeline Carr [9] points out, private business interests may not be aligned with the public good. When these businesses control critical public processes, as in Spain, it can be a barrier for the rest of the stakeholders to collaborate with public organisations.

This research has some limitations. First, while conclusions may be drawn from the conceptualisation of five different forms of capital, the variable construction in the Delphi questionnaire may have limited the validity of our results. For this reason, we supplemented the Delphi study results with in-depth interviews. Second, our network analysis can be biased by the experts’ selection who participated in the interviews because companies or organisations not mentioned by the experts were not displayed in the network map. Although we tried to overcome this limitation by including experts from all sectors, the inclusion of new experts could not have substantially changed the main result—i.e., the INCIBE’s and CCN’s more significant social capital. Nevertheless, our results should be tested in subsequent studies.

Cybersecurity has many facets. This paper developed an approach to explore cybersecurity governance in Spain, which should be complemented in future studies that delve deeper into how cybersecurity is provided in specific areas. For example, a plural policing approach should also be helpful when exploring cybersecurity governance in critical infrastructures in Spain, as previous research has already been done in other countries (e.g., [34]). Governments do not typically apply ‘one size fits all’ policies [40]. In this regard, Weiss and Jankauskas [41, p. 271] concluded that ‘the nature of the cybersecurity problem induces the choice of more or less hierarchy and thus control’.

Therefore, more research is needed to explore and compare critical infrastructure protection, the fight against cyberterrorism, cyberhate, disinformation campaigns, cyber-fraud, cyberbullying, and state-sponsored cyber operations. Some future research questions may be: to what extent different modes of network governance coexist depending on the nature of the cybersecurity problem? And, to what extent are the relevant actors similar and different depending on the cybersecurity problem? In this sense, financial institutions may likely play a preeminent role in the fight against cyber fraud. In contrast, social networking service companies such as Meta or Twitter may be more relevant in policing cyberhate. The knowledge generated by these future studies can help identify gaps in the governance of specific types of cybersecurity threats.

## Appendix

**Table 6:** List of 75 nodes identified by the 129 experts participating in round 1 as the most relevant for cybersecurity in Spain

Node	Mentions	%	Score
Spanish National Cybersecurity Institute (INCIBE)	119	92.2	0.70
National Cryptologic Centre (CCN)	114	88.4	0.72
National Centre for Infrastructure Protection and Cybersecurity (CNPIC)	50	38.8	0.21
Department of Homeland Security (DSN)	49	38.0	0.24
Joint Cyberspace Command	45	34.9	0.15
Civil Guard (GC)	26	20.2	0.11
Law enforcement agencies	24	18.6	0.08
National Police (UIT)	24	18.6	0.08
Telefónica	20	15.5	0.07
CSIRT (Spain)	14	10.9	0.05
Cybersecurity companies	12	9.3	0.03
Private companies	9	7.0	0.05
Spanish Data Protection Agency	8	6.2	0.02
National Cybersecurity Council	7	5.4	0.05
Deloitte	6	4.7	0.02
Hacker community	6	4.7	0.02
Indra	5	3.9	0.02
S21Sec	5	3.9	0.01
Universities	5	3.9	0.01
Cybercrime Prosecutor’s Office	4	3.1	0.02
ENISA	4	3.1	0.02
Corporate Information Security Departments	3	2.3	0.02
Industrial Cybersecurity Center (CCI)	3	2.3	0.01
Microsoft	3	2.3	0.01
National Security Council	3	2.3	0.01
Presidency of the Government	3	2.3	0.01
Spanish Secretary of State for Digitalisation and Artificial Intelligence	3	2.3	0.01
Citizens	2	1.6	0.01
Cybersecurity Agency of Catalonia	2	1.6	0.01
Defence Staff (Estado Mayor de la Defensa)	2	1.6	0.01
Eleven Paths	2	1.6	0.01
Financial institutions	2	1.6	0.01
Google	2	1.6	0.01
Information Systems Audit and Control Association (ISACA)	2	1.6	0.01
ISMS Forum Spain	2	1.6	0.01
McAfee	2	1.6	0.01
National Cybersecurity Forum	2	1.6	0.01
Red Iris	2	1.6	0.00
S2 Grupo	2	1.6	0.00
Spanish Cybersecurity Innovation Cluster (AEI Ciberseguridad)	2	1.6	0.00
Technology companies	2	1.6	0.00
Think tanks	2	1.6	0.00
Amazon	1	0.8	0.01
Apple	1	0.8	0.01
Army—Ministry of Defence	1	0.8	0.01
Autonomous police	1	0.8	0.01
Basque Cybersecurity Centre (BCSC)	1	0.8	0.01
CISO Group	1	0.8	0.01
Committee CTN 320—Cybersecurity and personal data protection	1	0.8	0.01
Communication infrastructure managers	1	0.8	0.01
Consultancy firms	1	0.8	0.01
Critical infrastructure managers	1	0.8	0.01
Cybersecurity Certification Agency (ACC)	1	0.8	0.00
General Security Directorates of Companies	1	0.8	0.00
Intelligence and Global Security Community (CISEG)	1	0.8	0.00
Interpol	1	0.8	0.00
Internet Segura for Kids (INCIBE)	1	0.8	0.00
Kaspersky	1	0.8	0.00
Local police	1	0.8	0.00
National Security Office	1	0.8	0.00
Office for Internet Security (INCIBE)	1	0.8	0.00
Public institutions	1	0.8	0.00
Red.es	1	0.8	0.00
RENIC—Spanish Network of Excellence on Cybersecurity Research	1	0.8	0.00
Santander Bank	1	0.8	0.00
Secretary-General of Digital Administration	1	0.8	0.00
Sector regulator (Bank of Spain, Ministry of Industry, DGS Insurance)	1	0.8	0.00
SEPROSE	1	0.8	0.00
Sophos	1	0.8	0.00
Spanish Standing Committee on Cybersecurity	1	0.8	0.00
State bodies	1	0.8	0.00
UNECE	1	0.8	0.00
United Nations	1	0.8	0.00
Virus total	1	0.8	0.00
Zerolynx	1	0.8	0.00

## Footnotes

1 We acknowledge that the impact of NIS2 Directive on Spanish cybersecurity policies and institutions was not covered by this research. Thus, future research should compare the current results with the developments produced by the NIS2 Directive.

2 The Spanish Constitution, Part IV “Government and Administration”, Article 97, states that “The Government directs domestic and foreign policy, civil and military administration and the defence of the State. It exercises executive and statutory authority in accordance with the Constitution and the law”.

3 The remaining offices can be consulted on the official website of the DSN, and they cover: (i) monitoring and warning, (ii) political and strategic planning, (iii) systems and infrastructure, (iv) crisis management and exercises, (v) homeland security analysis, and (vi) support and technical secretariat. The organization’s chart can be found here: https://www.dsn.gob.es/es/sistema-seguridad-nacional/departamento-seguridad-nacional.

4 The national cybersecurity system is regulated by Royal Decree 43/2021, which implements Royal Decree-Law 12/2018, on the security of networks and information systems, and, more recently, by Royal Decree 311/2022, regulating the National Security framework.

5 Operational bodies are those that instrumentally implement plans, programmes and policies, and deliver services to users in accordance with public policy. In the Spanish public administration, there are also political bodies (which are composed of elected officials responsible for drawing up policies, plans and strategies) and technical bodies (composed of personnel specialised in a particular subject whose objective is to advise the political bodies).

999 “STIC” stands for “*Seguridad de las Tecnologías de la Información y la Comunicación*” which in English means “information and communication technologies security”. The “CCN-STIC guides” are guides produced by the Spanish National Cryptologic Center with relevant information about ICTs security.

992 SAT stands for “*Sistemas de Alerta Temprana*” in the original version in Spanish, which can be translated as “Early-Warning Systems”. INET refers to the intranet of the organization. SAT INET describes the CCN service of individual probes offered to public organizations that can implement in their intranet to detect attack patterns and threats by analysing traffic and its flows. This is one of the three early-warning systems offered by the CCN along with the SAT ICS (for “Industrial Control Systems”) and SAT SARA (“*Sistema de Aplicaciones y Redes para las Administraciones*”, in English “Applications and Network System for Administrations”).

6 The national CERTs were regulated by Royal Decree-Law 12/2018 of 7 September on the security of networks and information systems and Royal Decree 43/2021 of 26 January implementing Royal Decree-Law 12/2018 of 7 September on the security of networks and information systems, which transposed the NIS Directive (Directive (EU) 2016/1148 of the European Parliament and of the Council of 6 July 2016 concerning measures for a high common level of security of network and information systems across the Union).

7 Specifically, the CCN-CERT services are ‘a) Support and coordination for the treatment of vulnerabilities and the resolution of security incidents that the entities within the scope of this Royal Decree have. The CCN-CERT, through its technical support and coordination service, shall act as quickly as possible in the event of any aggression received in the information systems concerned. In order to fulfil the purposes set out in the preceding paragraphs, reports, audit records and configurations of the systems concerned and any other information deemed relevant may be collected, as well as any computer media deemed necessary for the investigation of the incident of the systems concerned, without prejudice to the provisions of the applicable data protection regulations, as well as to the possible confidentiality of institutional or organizational data. b) Research and dissemination of best practices on information security among all members of public sector entities. To this end, the series of documents CCN-STIC (CCN Security of Information and Communication Technologies), prepared by the CCN, will provide standards, instructions, guides, recommendations and best practices to implement the ENS and to ensure the security of the information systems within the scope of this Royal Decree. c) Training for public sector staff specializing in information technology security to facilitate the updating of knowledge and to raise awareness and improve their capacities for the prevention, detection and management of incidents. Link to the official version: https://www.boe.es/eli/es/rd/2022/05/03/311 21 d) Information on vulnerabilities, alerts and warnings of new threats to information systems, collected from a variety of well-known sources, including their own’.

8 During this cyber crisis, the WannaCry ransomware affected several critical companies and hospitals in Spain. Among them, Telefónica, one of the major telecommunication companies in the country.

9 According to Royal Decree-Law 12/2018 of 7 September on the security of networks and information systems, article 16.

10 More information about the National Cybersecurity Forum can be found in https://foronacionalciberseguridad.es/.

11 For example, the Andalusian Cybersecurity Agency is expected to be established soon. See press note here: https://www.juntadeandalucia.es/organismos/presidenciainteriordialogosocialysimplificacionadministrativa/servicios/actualidad/noticias/detalle/260653.html.

## References

[1] Araluce G (2021) El Ciberataque al SEPE Se Produjo Tras Caducar Un Contrato de Mantenimiento Informático. Vozpopuli 06 (https://www.vozpopuli.com/espana/ciberataque-sepe-contrato.html.)

[2] Bederna Z, Rajnai Z (2022) Analysis of the cybersecurity ecosystem in the European Union. Int Cybersecur Law Rev 3(1):35–49. 10.1365/s43439-022-00048-9

[3] Boeke S (2018) National cyber crisis management: different European approaches. Governance 31(3):449–464. 10.1111/gove.12309

[4] Borgatti SP, Martin GE, Johnson JC (2013) Analyzing social networks. SAGE, Los Angeles

[5] Bourdieu P (1986) The forms of capital. In: Richardson J (ed) Handbook of theory and research for the sociology of education. Greenwood Press, New York, pp 241–258 (http://www.socialcapitalgateway.org/sites/socialcapitalgateway.org/files/data/paper/2016/10/18/rbasicsbourdieu1986-theformsofcapital.pdf.)

[6] Broeders D (2021) Private active cyber defense and (international) cyber security—pushing the line? J Cybersecur 7(1):tyab10. 10.1093/cybsec/tyab010

[7] Buil-Gil D, Miró-Llinares F, Moneva A, Kemp S, Díaz-Castaño N (2020) Cybercrime and shifts in opportunities during COVID-19: a preliminary analysis in the UK. Eur Soc. 10.1080/14616696.2020.1804973

[8] Burris S, Drahos P, Shearing C (2005) Nodal governance. Aust J Leg Philos 30:1–44. https://ssrn.com/abstract=760928

[9] Carr M (2016) Public-private partnerships in national cyber-security strategies. Int Affairs 92(1):43–62. 10.1111/1468-2346.12504

[10] Christou G (2016) Cybersecurity in the European Union. Palgrave Macmillan UK, London 10.1057/9781137400529

[11] Cohen J (1960) A coefficient of agreement for nominal scales. Educ Psychol Meas 20(1):37–46. 10.1177/001316446002000104

[12] Council of Europe (2001) Convention on Cybercrime. Vol. 185. https://www.coe.int/en/web/conventions/full-list/-/conventions/treaty/185. Accessed 12 July 2022

[13] Council of the European Union General Secretariat of the Council (2010) Estrategia de seguridad interior de la Unión Europea: hacia un modelo europeo de seguridad. Publications Office, LU (https://data.europa.eu/doi/10.2860/881)

[14] Dalkey NC (1969) The delphi method: an experimental study of group opinion. The RAND Corporation, Santa Monica

[15] Díaz-Fernández AM, Arcos R (2021) A framework for understanding the strategies of openness of the intelligence services. Int J Intell Secur Public Aff 23(3):259–280. 10.1080/23800992.2021.2010365

[16] Dirección General de Industria y de la Pequeña y Mediana Empresa (2019) Marco Estratégico En Política de PYME 2030. Ministerio de Industria, Comercio y Turismo, Madrid

[17] Do Vale HF (2021) Cuatro Décadas de Distribución Del Poder Territorial En España: Una Medición de La Autonomía Subnacional (1974–2018)/Four Decades of Territorial Distribution of Power in Spain: A Measurement of Subnational Autonomy (1974–2018). Rev Espanola Invest Sociol. 10.5477/cis/reis.173.3

[18] Dunn-Cavelty M, Suter M (2009) Public–private partnerships are no silver bullet: an expanded governance model for critical infrastructure protection. Int J Crit Infrastructure Prot 2(4):179–187. 10.1016/j.ijcip.2009.08.006

[19] Dupont B (2004) Security in the age of networks. Polic Soc 14(1):76–91. 10.1080/1043946042000181575

[20] Dupont B (2006) Delivering security through networks: surveying the relational landscape of security managers in an urban setting. Crime Law Soc Change 45(3):165–184. 10.1007/s10611-006-9033-5

[21] European Council (2010) The stockholm programme: an open and secure europe serving and protecting citizens. Off J Eur Union 2010/C 115/01. https://eur-lex.europa.eu/legal-content/EN/ALL/?uri=celex%3A52010XG0504%2801%29

[22] Freeman LC (1978) Centrality in social networks conceptual clarification. Soc Networks 1(3):215–239. 10.1016/0378-8733(78)90021-7

[23] International Telecommunications Union (2021) Global cybersecurity index 2020. Measuring commitment to cybersecurity. International Telecommunication Union, Geneva

[24] Johns E (2020) “Cyber security breaches survey 2020: statistical release.” department for digital, culture, media & sport. https://assets.publishing.service.gov.uk/government/uploads/system/uploads/attachment_data/file/893399/Cyber_Security_Breaches_Survey_2020_Statistical_Release_180620.pdf. Accessed: 28 March 2022

[25] Johns E (2021) “Cyber Security Breaches Survey 2021: Statistical Release.” Department for Digital, Culture, Media & Sport. https://assets.publishing.service.gov.uk/government/uploads/system/uploads/attachment_data/file/893399/Cyber_Security_Breaches_Survey_2020_Statistical_Release_180620.pdf. 28 March 2022

[26] Johnston L, Shearing C (2003) Governing security: explorations in policing and justice. Routledge, London, New York

[27] Leppänen A, Kankaanranta T (2020) Co-production of cybersecurity: a case of reported data system break-ins. Police Pract Res 21(1):78–94. 10.1080/15614263.2018.1525382

[28] Levi M, Williams ML (2013) Multi-agency partnerships in cybercrime reduction: mapping the UK information assurance network cooperation space. Inf Manag Comput Secur 21(5):420–443. 10.1108/IMCS-04-2013-0027

[29] Loader I, Walker N (2007) Civilizing security. Cambridge University Press, Cambridge ; New York

[30] Marsden PV (1982) Brokerage behavior in restricted exchange networks. In: Marsden PV, Lin N (eds) Social structure and network analysis. SAGE, pp 201–218

[31] Melander L, Dubois A, Hedvall K, Lind F (2019) Future goods transport in Sweden 2050: using a delphi-based scenario analysis. Technol Forecast Soc Change 138:178–189. 10.1016/j.techfore.2018.08.019

[32] Moneva A, Leukfeldt ER (2022) Insider threats among Dutch SMes: nature and extent of incidents, and cyber security measures 10.31219/osf.io/eqpb2 (OSF Preprints)

[33] Nøkleberg M (2020) Examining the how of plural policing: moving from normative debate to empirical enquiry. Br J Criminol 60(3):681–702. 10.1093/bjc/azz080

[34] Pomerleau P‑L, Lowery DL (2020) Countering cyber threats to financial institutions. A private and public partnership approach to critical infrastructure protection. Palgrave Macmillan 10.1007/978-3-030-54054-8

[35] Poster WR (2018) Cybersecurity needs women. Nature 555(7698):577–580. 10.1038/d41586-018-03327-w 10.1038/d41586-018-03327-w 29595805

[36] Quéro Y‑C, Dupont B (2019) Nodal governance: toward a better understanding of node relationships in local security governance. Polic Soc 29(3):283–301. 10.1080/10439463.2017.1391808

[37] Raskin MS (1994) The Delphi study in field instruction revisited: expert consensus on issues and research priorities. J Soc Work Educ 30(1):75–89. 10.1080/10437797.1994.10672215

[38] Spanish National Cybersecurity Institute (2022) “The national security council designates INCIBE as the national coordination centre of the European cybersecurity competence centre.” official press release. press room (blog). https://www.incibe.es/en/press-room/news/national-security-council-designates-incibe-national-coordination-centre-european. Accessed 29 Sept 2022

[39] van Stokkom B, Terpstra J (2018) Plural policing, the public good, and the constitutional state: an international comparison of Austria and Canada—Ontario. Polic Soc 28(4):415–430. 10.1080/10439463.2016.1205065

[40] Weiss M, Biermann F (2021) Cyberspace and the protection of critical national infrastructure. J Econ Policy Reform. 10.1080/17487870.2021.1905530

[41] Weiss M, Jankauskas V (2019) Securing cyberspace: how states design governance arrangements. Governance 32(2):259–275. 10.1111/gove.12368

[42] White A (2012) The new political economy of private security. Theor Criminol 16(1):85–101. 10.1177/1362480611410903

